# Comprehensive Evaluation and Optimization Model of Regional Fire Protection Planning of Major Hazard Sources Based on Multiobjective Fuzzy Theory

**DOI:** 10.1155/2022/3517836

**Published:** 2022-02-11

**Authors:** Fujiang Chen, Junying Chen, Jingang Liu

**Affiliations:** ^1^Xihua University, School of Emergency Management, Chengdu 610039, Sichuan, China; ^2^China Railway Scientific Research Institute Co., Ltd., Chengdu 610032, Sichuan, China

## Abstract

The regional fire protection plan is an important part of the city's overall plan, which represents the deepening of the regional fire protection plan and the specific content of the city's economic regional fire protection plan. Fire protection is an important part of national economic and social development, and it is also one of the indicators to measure the level of modern culture. Effective and practical fire protection planning can effectively prevent and reduce fire risks and protect the lives and property of the people, which is very important for social development. In order to optimize the regional fire model, this study uses a very objective fuzzy theory to analyze and discuss the research objects. In view of the large amount of fuzzy information in fuzzy optimization, fuzzy criterion recognition, and fuzzy grouping, based on the generalized fuzzy distance and fuzzy number, this paper proposes a multiobjective fuzzy theory-based comprehensive evaluation and optimization model of fire planning for major hazard sources. The results show that hotel fire risks tend to be higher, which is the focus of people's attention. Among the fire hazards, wholesale, retail, and catering industries are the hardest hit areas, accounting for 66.3%, and some other industries are also disaster areas that need to be dealt with.

## 1. Introduction

### 1.1. Background and Significance

With the rapid development of modern social economy and technology, and the great change of energy structure, focus has changed from relying mainly on coal to relying more on the production of petrochemical products. Due to the popularity of the petrochemical industry, many related companies have come here. Petrochemical industry is the cornerstone of national economic development, related to social production and people's life, and plays an important role in promoting social and economic development and improving people's living standards. However, the petrochemical industry is also a very dangerous industry. The accumulation of dangerous and harmful substances has the characteristics of flammability, explosion, high temperature, high pressure, toxic, and harmful. Petrochemical enterprises must pay special attention to fire safety management in production and operation. If a fire occurs in a certain area, other areas will be affected, resulting in an increase of accident impact and the expansion of the disaster area, which will lead to serious economic loss, social loss, and social consequences. So, it is particularly important to carry out fire planning in high-risk areas (those containing flammable, explosive, high temperature, high pressure, toxic, and harmful items).

### 1.2. Related Work

Fire station layout planning is an important part of fire protection system. At present, there are many unreasonable and subjective factors in the latheyt planning of domestic fire stations. Wenjuan used the optimization theory design to establish a reasonable framework for the optimization model of the fire station. He transformed the multiobjective fuzzy theory into a unified single-objective model and determined the regional priority and danger level of each part of the fire according to the fire risk assessment method and transformed it into a genetic algorithm to solve the model [1].

There are always some problems in the mountain fire station. H. Ren takes the planning of the mountain fire station as the research object, mainly aiming to address the shortcomings of the mountain fire planning and the new problems brought about by the rapid development of the city, and identifies and evaluates the fire risk of the buildings in the mountain city. On the basis of fire risk assessment, GIS technology is introduced to solve the problems of location selection of new fire station and latheyt of old fire station. In an area proximal to high fire incidence area, 4 fire stations shall be added. At the same time, the new fire stations are also required to be close to the urban main road as much as possible. The comprehensive application of various methods provides scientific basis and suggestions for the latheyt optimization of municipal fire stations [2].

In the traditional aviation forest protection, the work of cruise and fire location using topographic map and GPS is heavy and difficult, and the time of forest and fire investigation is limited. Jiang analyzed the application of Google Maps in aerial forest protection work. This method is conducive to improving the attention of flight observers in positioning navigation and forest fire investigation and improving the efficiency of aerial forest protection. This paper introduces the functions of Google Earth software in searching destination; acquiring 3D terrain; acquiring landmark; adding annotation; and measuring length, perimeter, and area, and discusses the simulation application of Google Earth, including route planning, firepower positioning, surrounding terrain prediction, etc., according to route navigation, and drawing fire behavior map and building forest fire information database [3].

### 1.3. Innovation

The purpose of this study was to make a reasonable and effective plan for the regional fire control of major hazard sources, so as to reduce personal injury and economic loss caused by fire accidents. Using the theory of fuzzy set to evaluate the fire risk can provide reasonable early warning for fire protection in major hazard areas. The results of the study of a hotel fire building show that the fire risk of the hotel belongs to a higher risk, accounting for 17% of all fire accidents.

## 2. Related Concepts

### 2.1. Multiobjective Fuzzy Theory of the Fire Protection System

In the design of the fire protection system, the models used for fire station latheyt belong to the category of maximum coverage model. The most typical is the problem of MCLP [4]. This model limits the optimal address of gas station to the network node and gives the optimal algorithm in general. The purpose of this model is to find the largest cell group that a server can serve in a normal time or space when the number of servers is limited [5, 6]. However, this model does not consider server congestion and assumes that there are always inactive servers when providing services. In addition, the parameters and structure of these constraint models can be determined [7].

For the fire protection design model, some parameters are not clear and are uncertain, and each part of the whole system has different rules, which means that the objective design problem should be used for analysis and modeling [8]. For example, the time or distance required from a fire station to the scene of a fire is a very important factor in limiting or targeting the function of the MCLP model. However, the traditional MCLP model only deals with time simply, and does not fully consider the difference of time. The degree of fire danger will greatly affect the scientific nature of the site selection decision [9, 10]. This is because different levels of fire need different times to put out the fire. At the same time, the demand points of different risk levels have different fire rescue levels. In the model [11], it is very important to reflect the individual differences between demand points for the optimal design of fire stations. Therefore, conducting a global study requires multiobjective planning [12]. Global research means a thorough study of the fire scene to detect and prevent fires in the first place.

The basic definition of Fuzzy Theory:

If *X* represents the target set of *x*, fuzzy set A represents an ordered queue set.

(1) $$\begin{array}{c} A=\left\{\left(x,\mu_{A}∼\left(x\right)\right)|x\in X\right\}. \end{array}$$

Here, *x* represents the element in set *X*, and *μ*_A_ ~ (*X*) represents the membership function or membership degree of element *X* in fuzzy set *A*, the membership space of mapping *x* is [0, l].

If

(2) $$\begin{array}{c} \mu_{\text{A}}∼\left(\lambda x_{1}+\left(1-\lambda \right)x_{2}\right)\geq \min\left\{\mu_{\text{A}}∼\left(x_{1}\right),\mu_{\text{A}}∼\left(x_{2}\right)\right\},x_{1},x_{2}\in X,\lambda \in \left[0,1\right], \end{array}$$

then fuzzy set A is convex. If sup_*x*_*μ*_*A*_(*x*) ≈ 1, then fuzzy set A is called a normal set. The membership function *μ*_c_ ~ (*x*) of intersection C of fuzzy sets A and B are as follows:

(3) $$\begin{array}{c} \mu_{\text{C}}∼\left(x\right)=\min\left\{\mu_{\text{A}}∼\left(x\right),\mu_{\text{B}}∼\left(x\right)\right\},x\in X,\\ \mu_{\text{C}}∼\left(x\right)=\mu_{\text{A}}∼\left(x\right)\mu_{\text{B}}∼\left(x\right),x\in X. \end{array}$$

So far, the introduction to fuzzy sets is completed.

### 2.2. Regional Risk Classification

According to the scope of fire risk and basic characterization, regional risk is divided into regional fixed risk and regional actual risk [13]. Regional fixed risk refers to the impact caused by regional risk and is one of the main sources of accident risk. The actual regional risk is based on the internal risk of the region, taking into account the extension or inhibition of regional factors that affect the risk of accident consequences. The combination of regional risk factors and internal risk indicators is an indicator system to evaluate regional reality [14].

#### 2.2.1. Regional Risk Assessment

Regional fixed risks are mainly caused by regional accidents, and inherent risks can be quantified by specific methods [15]. At present, the commonly used quantitative analysis methods include fire and explosion index method, six stage risk assessment method, Monde assessment method, inflammable material assessment method, and major hazard source assessment method [16].

#### 2.2.2. Factors Affecting Regional Risk

Regional risk factors can be divided into risk diffusion factors and risk mitigation factors. Risk diffusion factors refer to the factors that enhance the impact of accident consequences, especially economic vulnerability and social sensitivity [17]. Economic vulnerability refers to the impact on buildings, mechanical devices, residential areas, etc. In post-accident areas, this is usually measured by economic loss indicators [18]. Social sensitivity is often related to psychology, which refers to the psychological damage caused by panic caused by accidents.

Risk mitigation factors can mitigate accident consequences and enterprise emergency management level. The government is an important factor to reduce accident risk [19]. If there is no accident, the assessment of emergency management level is often reflected in the construction of an emergency team and the formulation of an emergency plan. In the case of an accident, the content of emergency management level measurement is mainly the protection of emergency resources available at the scene of the accident and the protection of resources, which is finally reflected in the emergency rescue and evacuation capacity of the area [20].

### 2.3. Fire Risk Assessment Method

After long-term development and improvement, the fire risk assessment method has developed to more than 40 kinds, and has been widely used globally. Various quantitative expression forms of evaluation results can be divided into three evaluation methods: quality evaluation method, semiquantitative evaluation method, and quantitative evaluation method [21].

Quality evaluation is the basis for improving product quality. The establishment of a complete quality evaluation system can predict the development trend of the macro quality level; improve the market competitiveness of enterprises; prevent potential dangers and threats to human health, safety, and living environment; and enable people to exchange products, services, and resources with each other. Choose adequately and reasonably in a market economy. The quality assessment method is based on industry standards, regulations, rules, etc., allowing assessment experts with rich theoretical and practical knowledge to draw conclusions based on their observation and experience. The conclusion should consider the following conditions: if there are substances that may cause danger in the area, whether the management within the scope meets the requirements, and the possibility and scope of risk occurrence. The whole evaluation is mainly based on expert reasoning and intuitive judgment, and the mathematical model on which the theory is based is generally simple. This evaluation method has the advantages of simple operation and intuitionistic evaluation conclusion, and has been widely used. However, due to the lack of standards and subjectivity of evaluators in the process of operation, the conclusions drawn are quite different [22, 23].

The semiquantitative evaluation method should identify and distinguish the potential risk factors in the evaluation target according to the risk degree. The basis is: the corresponding classification standard, which is used to classify the determined factors, determines the relevant evaluation indexes, and establishes a link. The link can be a comprehensive mathematical model that defines the relative values of patience and overall safety parameters and quickly assesses the risk level of the object. Semiquantitative method makes up for the shortcomings of randomness and difference of quality method, but it is still simple and intuitive. Fire and explosion indicator method, Monde method, Japan's six stage assessment method, and China's risk classification method are the most commonly used semiquantitative assessment methods.

The quantitative evaluation method includes problem identification, risk identification, cause analysis and impact assessment, risk assessment, and final assessment conclusion. The purpose of the quantitative method is to get more accurate evaluation results. The quantitative evaluation method should be based on the quantification of the evaluation index system. All risk assessment indicators should be consolidated and quantified in accordance with specific criteria. Use a specific mathematical model. Get the risk value of the evaluation target. When analyzing a relatively complex system, the quantitative evaluation method can more clearly reflect the hierarchical relationship between different indicators. The conclusion drawn by this method is very intuitive and clear. We can easily compare the evaluation results of different subjects, or we can compare the evaluation results with the minimum social security standards, which is acceptable to people.

### 2.4. Develop Effective Plans for Fire Protection in Major Hazardous Areas

#### 2.4.1. The Establishment of Optimization Model of the Fire Water Supply System

In order to ensure that the fire-fighting system provides effective fire-fighting measures and reduces the losses caused by the fire, the design of the fire water supply system is a vital link in the fire protection planning. Whether the regional fire-fighting water supply system is designed to be reliable, reasonable, and economical will affect the scientific nature of the entire regional fire-fighting plan [24]. It can be seen from the previous norms and standards that the existing fire-fighting water consumption norms are mainly based on the population size of the city to determine the frequency of fires, thereby determining the fire-fighting water consumption of the city. However, for the design of regional fire protection planning, this kind of fire water consumption index based solely on population size is extremely unreasonable. For example, for an industrial area that contains some major hazards like liquefied petroleum gas tanks, the population may be relatively small in the area, but once a fire occurs in this area, the amount of fire extinguishing water required will be greater than that of a pure living area with the same population [25, 26]. Therefore, if the population of an area is solely used as the basis for determining the water volume of the fire water supply system, the design standard will be far lower than the water required for the actual fire, which will not effectively achieve the purpose of disaster reduction. Therefore, in order to comprehensively analyze the influencing factors of regional fire water consumption and use the interrelationship of various factors to plan the fire water consumption of the entire regional water supply system, it is necessary to adopt suitable optimization methods to establish a reasonable mathematical model of the fire water supply system. Specific influencing factors include water consumption, distance from the fire station, water storage, floor height, and flow of people. Its purpose is to realize the scientific rationality of the planning of the fire water supply system to meet the needs of actual production and life to achieve the purpose of disaster reduction and elimination.

#### 2.4.2. The Latheyt of Each Functional Block Is Reasonable

It should be noted that the fire hazard in the LPG operation area is high and there are many accidents. Once a fire occurs, it is extremely destructive. Although the fire accident in the LPG operation area may not directly affect the national strategic oil reserve base, it directly affects the petrochemical operation area, has an indirect impact on the national strategic oil reserve base, and increases the overall fire risk. From the perspective of the overall situation and long-term interests, it is possible to consider limiting the scale of LPG operation areas.

#### 2.4.3. Fire Station Latheyt

One of the main responsibilities of the fire department is to fight fire and rescue. This task usually comes from the arranged fire station. The fire station is the basic organization unit of the fire department for fire-fighting and rescue. The purpose of planning a fire station is to enable the fire station to provide effective fire-fighting and rescue services. Based on the analysis of the current situation of the fire protection environment, the number, location, area of responsibility, floor space, building area, vehicles, equipment, and personnel to be planned for the construction of fire stations are clearly defined, and the time to achieve these goals are determined. There are specific plans for human and material resources [27]. The planning of the fire station must adapt to the needs of multifunctional and large-area operations. For areas with special fire protection requirements, a fire station capable of handling special fires and disasters should be planned and established. If possible, the three-dimensional latheyt of water, land, and air should be considered for comprehensive development [28].

## 3. Experimental Design

### 3.1. Research Object

As a hotel has a large flow of people, a lot of flammable materials, and high floors, improper management can easily lead to huge fires. Once a fire occurs, it is a huge accident. So in this study, a hotel on fire is selected as the object, and the fuzzy set theory is used to evaluate the fire risk. The evaluation index system includes influencing factors, key variables, and hierarchy system. The dynamic and fuzzy characteristics of the system are reflected in the evaluation of fire risk, fire resistance, and fire vulnerability. In addition, this method is also a kind of evaluation method, which is widely used, practical, and verified by a large number of practices.

### 3.2. Research Steps

The fuzzy evaluation method has been widely used in fuzzy system evaluation for its good practicality. It uses an ambiguous theory and examines the use of multiple indicators to assess participation. Three key groups participated in the whole process, including a set of systematic factors, a set of systematic reviews, and a set of evaluation factors. Among them, the evaluation factors have the greatest influence and ultimately affect the evaluation results of credibility. According to the fire risk assessment index system, the system factor set *U*={*U*_1_, *U*_2_, *U*_3_}, wherein U1 represents the fire risk degree, U2 represents the fire vulnerability degree, and U3 represents the fire resistance degree. Determine the weight of system coefficient; each factor includes a set of evaluation indexes, and the total weight A of this factor is determined according to the weight of evaluation index a1, wherein *A*={*a*_1_, *a*_2_, *a*_3_}, ∑_*i*=1_^*n*^*a*_1_=1.

According to the fire protection level, it is divided into five evaluation levels. The evaluation set *V*={*V*_1_, *V*_2_, *V*_3_, *V*_4_, *V*_5_}, V1 is very safe, V2 is relatively safe, V3 is general safety, V4 is relatively dangerous, and V5 is very dangerous. The specific evaluation process is as follows:

Through 10 experts to grade the second-level indicators under the first-level indicators, grade and summarize the second-level indicators under the fire risk U1 of a hotel. The fuzzy subsets of fire risk can be obtained, and then U2 fire vulnerability and U3 are the fuzzy subsets A2 and A3 of fire resistance:

1. These experts need to score the first-level indicators of fire risk U1, fire vulnerability U2, and fire resistance U3, and synthesize the results,
2. The fuzzy subset *A*={*A*_1_, *A*_2_, *A*_3_} of fire risk factors is calculated by using the formula.
3. Factor evaluation. 30 experts were invited to grade the indicators in the indicator system according to the specific conditions of the experimental subjects and the criteria of indicator quantification. For example, for an indicator, 13 experts think it is very safe, 11 experts think it is safe, and 6 experts think it is safe. Then, the evaluation set of this factor is defined as {0, 47, 0, 42, 0, 11, 0, 0}. The sum of all evaluation sets is 1. This rule also applies to other indicators, and then summarizes the evaluation value of each individual factor.
4. *R*_1_=*A*_1_*∗r*_1_, *R*_2_=*A*_2_*∗r*_2_, *R*_3_=*A*_3_*∗r*_3_, get the fuzzy matrix *R*={*R*_1_, *R*_2_, *R*_3_}, through the formula *B*=*A∗R*, according to the scoring of experts, finally determine the fire risk level.

## 4. Regional Fire Protection Planning of Major Hazard Sources

### 4.1. Fire Risk Assessment

The fire risk assessment process is closely related to the fire development process. In general, the specific development process can be divided into the following processes: fire, growth, full development, recession, and finally extinction. In the above experiment, a hotel is taken as the object, and the fuzzy set theory is used for fire risk assessment and analysis. At the same time, the level of fire risk assessment in recent years can be found by querying the National Statistical Yearbook. The results are shown in Table 1.

According to the data provided in Table 1, we can clearly see that relative safety accounts for half of the fire risk rating of the experts. In fire vulnerability, most experts choose the fire is very safe. In the fire resistance industry, most experts give the scores of very safe and relatively safe. It can be seen that the fire risk is the most likely factor to increase the fire risk, followed by fire vulnerability and fire resistance.

### 4.2. Response Time of Fire Accidents

Using many objective and uncertain methods to solve the problem of optimizing the latheyt of fire stations has a simple feature: adaptability. Combined with the analysis and research on the level of fire risk assessment and the reference statistics of some countries, the fuzzy characteristics can be better determined. As shown in Table 2, it is the recommended fire response reference value of the fire protection system.

The rescue time of fire-fighting is calculated in seconds. The earlier the rescue team arrives at the scene of the accident, the faster the control of the accident will be. Table 2 also shows that different risk levels have different response times. The average operating speed can be used to turn the requirement of operating time into a distance limit. Risk levels should be used to distinguish multiple limits on the distance from the fire station to the scene of the accident. The calculation of this risk level should be based on the statistics of different regions. Fire risk assessment and analysis can be used to obtain data required for different risk levels. The number of fire vehicles and firefighters shall be determined according to the latest regional statistical accident report and fire station budget.

### 4.3. Data Analysis of Major Hazard Accidents

Relevant data show that in 2007, five of the top ten fires outside China occurred due to major flammable and explosive hazards in urban public places and facilities. Although the causes and consequences of accidents are quite different, major accidents involving hazardous chemical sources have some common characteristics: we ought to prevent accidents, which not only leave a large number of casualties inside and outside the factory but also cause huge property losses, and eventually damage the environment with immediate effect. At present, China has not established a systematic and effective major risk supervision system; the latheyt, category, and dynamic status of major hazard sources are unclear; and the ability of supervision, control, early warning system, and emergency rescue to prevent emergencies has not been established, so major accidents happen repeatedly.

**Figure 1:**
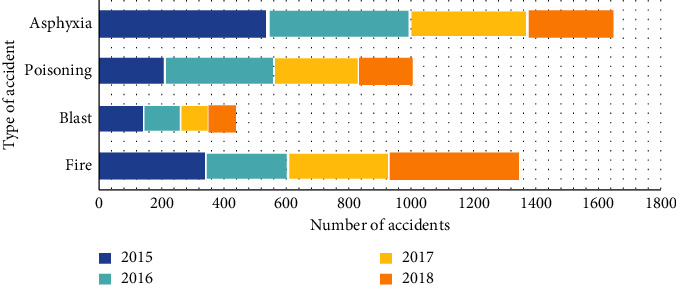
Hazard area accident.

As shown in Figure 1, major safety accidents occur frequently. The main reason is that the location of major hazard sources in most cities is not planned, the classification is not clear, the effective supervision system of major risk sources cannot be established, the emergency rescue plan cannot be determined, and the demand and ability of emergency dispersion cannot be determined. In particular, the goal of macro-control management is not clear. Therefore, how to avoid the occurrence of major accidents and how to implement regular protective measures are the key issues in realizing emergency rescue. It is necessary to strengthen the awareness of fire control and implement fire control measures, and ensure timely and effective treatment for major accident victims.

### 4.4. Characteristics of Major Hazard Industries

The research field of major hazard sources is defined within the scope of cities.

**Figure 2:**
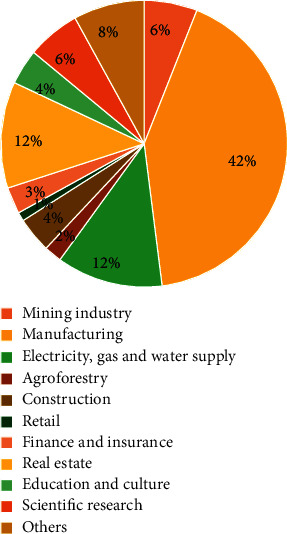
Characteristics of major hazard industries.

Typical units of major risk sources are production sites (such as chemical companies and heating facilities companies) and storage sites (such as oil, explosives, and toxic substances storage), which are distributed in areas in different urban buildings (such as gas filling stations and LPG station). Because the direct correspondence between important risk sources and land types is not easy to obtain, the main risk sources of land use types can be obtained only by defining the characteristics of important risk sources in the industry and the relationship between major risk industries and land use types (Figure 2).

### 4.5. Fire Water Consumption

In order to effectively implement fire rescue, it is necessary to evaluate the initial water consumption of each fire hazard source. Only by using the strong theoretical basis determined by the estimation of fire water consumption, can we truly plan a reasonable fire water supply system.

**Figure 3:**
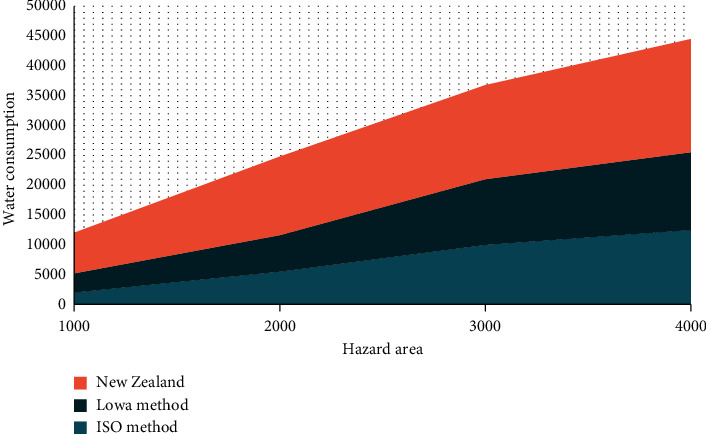
Fire water consumption comparison.

As shown in Figure 3, the calculation results of water consumption will be different for different estimation methods. Among them, the water consumption calculated by the New Zealand method is the most effective than that calculated by the other two methods, namely, the lowa method and ISO method.

### 4.6. Regional Fire Protection Planning

As a typical regional disaster causing factor, the main risk sources have different effects on regional risk. The distribution characteristics of the main risk sources are not equal to the spatial distribution, and in the absence of geographic information, they do not even belong to the category of spatial distribution. This study considers that major hazard sources have important characteristics in industrial distribution and urban land-use distribution. Regional risks are to be analyzed from this perspective. Especially when analyzing the main risk distribution characteristics of urban land, in addition to considering its own land, the distribution characteristics of different types of land affected by major risks should be fully analyzed according to different urban models. All urban land use information can be considered as major disaster source.

**Figure 4:**
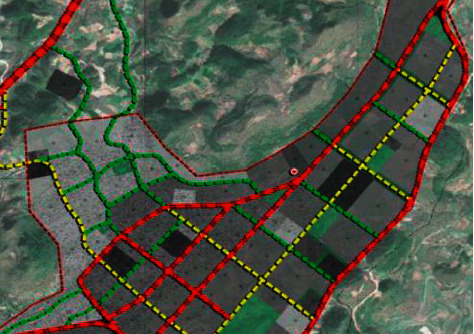
Regional fire protection planning.

As shown in Figure 4, in the absence of regional details, the main distribution of major risk sources should be adapted to the main land-use types and their characteristics. Combined with the characteristics of urban latheyt, especially the characteristics of land latheyt, preliminarily analyze the impact of major risks on different regions, divide the site into different types of land, and analyze the land types that may be affected by major risks. After the sites on various types of land are adjusted, the vulnerability analysis of these people's activity sites can be used as a perspective to analyze the risk distribution in the region. Setting up fire-fighting facilities in places with high risk can greatly reduce the destructive power of fire.

## 5. Conclusions

Multiobjective fuzzy decision-making is still a booming research field, and there are many theoretical and practical problems to be solved. The main work of this study focuses on the multiobjective fuzzy theory and its related fields, and some research results have been achieved. At the same time, there are many projects that need to be further studied and improved. The qualitative, objective, and weight of fuzzy set theory is one of the main research contents, which is based on the consistency of a series of projects and objectives. In most cases, this makes sense. However, there may be two other situations in the decision-making process. First, the priority given by the decision-maker does not conform to the principle of consistency; second, the decision-maker cannot give sufficient ranking. In other words, the sequencing of some projects or goals is incomplete or unknown. The fuzzy optimization model is applied to group decision-making, which further widens the application field of fuzzy sets, but at the same time, the aggregation of group fuzzy preferences also faces the problem of ordering consistency, that is, in addition to the inconsistency of individual preferences, there is also the inconsistency of group preferences. It is not only an interesting and realistic problem but also a common problem in group decision-making.

This paper is based on the multiobjective fuzzy theory, a comprehensive evaluation and optimization model for the fire protection planning of major dangerous source areas. The establishment of this model firstly analyzes the various influencing parameters of the fire station layout. The model not only considers conventional response time constraints but also economic constraints such as fire protection investment, disaster mitigation losses, and even all possible future situations. The waiting time of the fire station service and the major hazards that need to be considered make the layout optimization strategy of the regional fire station more suitable for the development needs of the society in the future. Based on national standards and successful cases abroad, this paper adopts multiobjective fuzzy planning theory and establishes a regional fire water supply system planning model based on major hazards. In the model, the area is first divided into several functional blocks. Calculate and analyze the fire-fighting water consumption of each functional block, and then consider the special requirements of major hazard sources of fire-fighting water consumption and the premise that the water supply system of each functional area can cooperate and assist, and then determine the fire-fighting water from the overall consideration of the area, in order to plan out the fire water supply system of the area.

According to the data of fire risk, fire resistance, and fire sensitivity, this study determines the state of disaster, analyzes the characteristics of major hazard sources, carries out risk assessment and emergency device investigation, analyzes the impact of chemical hazard sources on the site, and evaluates and optimizes the spatial latheyt of emergency sites. Detailed inspection of traffic conditions, land use, land characteristics, fire-prone areas, and other factors shall determine the candidate locations for fire planning latheyt. From the two levels of the government and the individual, the fairness goal, the economic cost goal, and the distance cost goal of the fire planning latheyt are set up, and the corresponding decision-making model is constructed.

## Figures and Tables

**Table 1:** Fire risk assessment.

Fire risk	Very safe	Relatively safe	General safety	More dangerous	Very dangerous
Fire hazard	7	15	8	0	0
Fire vulnerability	12	8	7	3	0
Fire resistance	16	12	2	0	0

**Table 2:** Fire accident response time.

Risk level	Time limit to reach the accident location	Distance from the accident site (km)	Response time to fire accidents (s)
I	3–6	3–6	35.5
II	6–10	6–10	33.2
III	11–13	11–13	29.6
IV	14–20	14–20	15.4

## Data Availability

The data that support the findings of this study are available from the corresponding author upon reasonable request.
